# Epidemiology and disease characteristics of systemic sclerosis-related pulmonary arterial hypertension: results from a real-life screening programme

**DOI:** 10.1186/s13075-017-1250-z

**Published:** 2017-03-07

**Authors:** Kathleen Morrisroe, Wendy Stevens, Joanne Sahhar, Candice Rabusa, Mandana Nikpour, Susanna Proudman, the Australian Scleroderma Interest Group (ASIG), Catherine Hill, Sue Lester, Peter Nash, Gian Ngian, Mandana Nikpour, Susanna Proudman, Maureen Rischmueller, Janet Roddy, Joanne Sahhar, Wendy Stevens, Gemma Strickland, Vivek Thakkar, Jenny Walker, Jane Zochling

**Affiliations:** 1 Department of Medicine, The University of Melbourne at St Vincent’s Hospital (Melbourne), 41 Victoria Parade, Fitzroy, 3065 Victoria Australia; 2 Departments of Rheumatology and Medicine, The University of Melbourne at St Vincent’s Hospital (Melbourne), 41 Victoria Parade, Fitzroy, 3065 Victoria Australia; 3 Monash University and Monash Health, 246 Clayton Road, Clayton, 3168 Victoria Australia; 4 Rheumatology Unit, Royal Adelaide Hospital, North Terrace, SA 5000 Australia; 5 Discipline of Medicine, University of Adelaide, Adelaide, SA 5000 Australia

**Keywords:** Systemic sclerosis, Scleroderma, Pulmonary arterial hypertension, Screening algorithm

## Abstract

**Background:**

Pulmonary arterial hypertension (PAH) is the leading cause of death in systemic sclerosis (SSc). Annual screening with echocardiogram (ECHO) is recommended. We present the methodological aspects of a PAH screening programme in a large Australian SSc cohort, the epidemiology of SSc-PAH in this cohort, and an evaluation of factors influencing physician adherence to PAH screening guidelines.

**Methods:**

Patient characteristics and results of PAH screening were determined in all patients enrolled in a SSc longitudinal cohort study. Adherence to PAH screening guidelines was assessed by a survey of Australian rheumatologists. Summary statistics, chi-square tests, univariate and multivariable logistic regression were used to determine the associations of risk factors with PAH.

**Results:**

Among 1636 patients with SSc, 194 (11.9%) had PAH proven by right-heart catheter. Of these, 160 were detected by screening. The annual incidence of PAH was 1.4%. Patients with PAH diagnosed on subsequent screens, compared with patients in whom PAH was diagnosed on first screen, were more likely to have diffuse SSc (*p* = 0.03), be in a better World Health Organisation (WHO) Functional Class at PAH diagnosis (*p* = 0.01) and have less advanced PAH evidenced by higher mean six-minute walk distance (*p* = 0.03), lower mean pulmonary arterial pressure (*p* = 0.009), lower mean pulmonary vascular resistance (*p* = 0.006) and fewer non-trivial pericardial effusions (*p* = 0.03). Adherence to annual PAH screening using an ECHO-based algorithm was poor among Australian rheumatologists, with less than half screening their patients with SSc of more than ten years disease duration.

**Conclusion:**

PAH is a common complication of SSc. Physician adherence to PAH screening recommendations remains poor. Identifying modifiable barriers to screening may improve adherence and ultimately patient outcomes.

**Electronic supplementary material:**

The online version of this article (doi:10.1186/s13075-017-1250-z) contains supplementary material, which is available to authorized users.

## Background

Systemic sclerosis (SSc) is a multisystem connective tissue disease (CTD) characterised by vasculopathy and fibrosis [1]. There is no cure for SSc, leading to significant morbidity, mortality and poor health-related quality of life [2, 3].

Cardiopulmonary manifestations, namely interstitial lung disease (ILD) and pulmonary arterial hypertension (PAH), have replaced SSc renal crisis (SRC) as the leading cause of mortality in SSc [4, 5].

SSc-PAH occurs with a prevalence of 8–12% [6] and is the second most common cause of PAH after idiopathic PAH [7]. PAH is characterised by abnormal proliferation, vasoconstriction and thrombosis of the pulmonary vasculature, leading to elevated pulmonary vascular resistance (PVR), resulting in right-heart failure and death [8]. Despite new pulmonary vasodilator therapies that improve symptoms and survival [9–16], one-year and three-year survival remains poor (78% and 47%, respectively) [17, 18], less than that of idiopathic and other connective tissue disease (CTD)-associated PAH [18]. Risk factors for the development of SSc PAH include anti-centromere antibody, telangiectasia, calcinosis, oesphageal stricture, sicca symptoms, mild ILD and digital ulcers [19, 20], although none of these perform sufficiently well as indicators in the individual patient and are often inconsistent between studies.

Early recognition of SSc-PAH is difficult as early disease is clinically silent and the heterogeneous nature of SSc makes interpretation of fatigue and dyspnoea challenging [21]. Survival is improved even after adjustment for lead-time bias in SSc-PAH when diagnosed by screening compared with diagnosis during routine care [9–14]. Consequently, annual screening with transthoracic echocardiogram (TTE) and pulmonary function tests (PFTs) is recommended [22], regardless of the presence or absence of the aforementioned risk factors, to identify patients who should undergo right-heart catheterisation (RHC) to confirm the diagnosis.

Despite the documented benefit of PAH screening, physician adherence is suboptimal, with one study showing that only 34.7% of patients with SSc had a TTE and 53.1% had PFT in the twelve months prior to PAH diagnosis [23]. Adherence among Australian physicians is no better, with over 40% not adhering to annual screening and even fewer using RHC for PAH diagnosis [24]. Consequently, the Australian Scleroderma Cohort Study (ASCS), a longitudinal multi-centre study, was established in 2007 as a web-based screening platform for the cardiorespiratory manifestations of SSc.

In this paper, we present the methodological aspects of establishing the ASCS and the screening algorithm, the characteristics of patients with SSc with PAH in this cohort and an evaluation of the factors influencing Australian rheumatologists’ adherence to screening guidelines.

## Methods

### Study centres

The ASCS is a nationwide project wherein patients with SSc are recruited from 13 participating centres across Australia. SSc experts were invited by a core group of rheumatologists to form the Australian Scleroderma Interest Group (ASIG) and to take part in the ASCS through recruitment of patients and collection of data at their centres. Physicians who are not at an ASIG centre and care for patients with SSc are invited to refer patients for the screening service, while ongoing care between screening visits remains their responsibility.

### Patients

All patients with SSc, defined according to American College of Rheumatology (ACR)/European League Against Rheumatism (EULAR) criteria [25] or Leroy/Medsger criteria [26], and mixed connective tissue disease (MCTD), as originally described by Sharp et al. [27], are eligible for enrolment. Patients provide written informed consent for collection of de-identified data, chart review, and storage of serum and DNA for future studies. All human research ethics committees of the participating sites have approved ASCS.

### Data collection and database

Comprehensive demographic and disease-related data are collected annually and entered into a custom-made database hosted by St. Vincent’s Hospital Melbourne using a remote secure access token. A log is kept of all users and the database is backed up daily. Logic checks are used to detect errors in data entry and incomplete entries.

### Screening algorithm for early detection of PAH

**Fig. 1:**
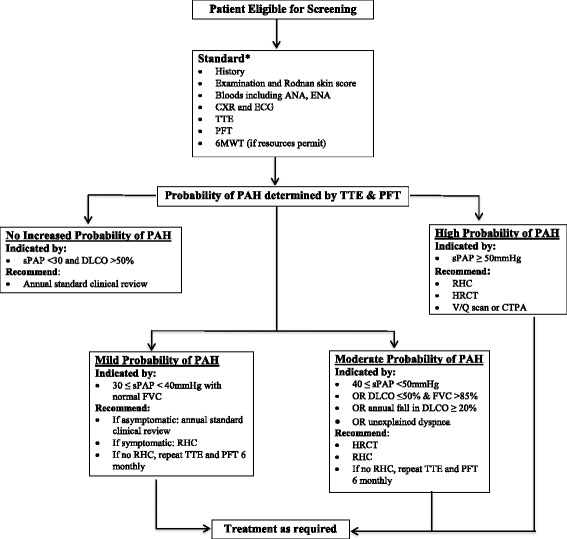
Australian Scleroderma Cohort Study algorithm for screening for pulmonary hypertension. *ANA* antinuclear antibody, *ENA* extractable nuclear antibody, *CXR* chest radiograph, *ECG* electrocardiogram, *TTE* transthoracic echocardiogram, *PFT* pulmonary function test, *6MWT* six-minute walk test, *PAH* pulmonary arterial hypertension, *sPAP* systolic pulmonary arterial pressure, *DLCO* diffusing capacity of the lung for carbon monoxide corrected for haemoglobin, *RHC* right-heart catheterization, *HRCT* high-resolution chest computed tomography, *V/Q* ventilation perfusion, *CTPA* computed tomography pulmonary angiography

Participation in the ASCS mandates annual application of a PAH screening algorithm created by a panel of Australian rheumatologists, cardiologists and respiratory physicians (Fig. 1) based on the European Society of Cardiology (ESC)/European Respiratory Society (ERS) guidelines [28, 29]. According to Fig. 1, investigations are recommended for symptomatic patients with low probability of PAH, and irrespective of symptoms for patients with moderate or high probability of PAH [30].

### Clinical decision support tool

If any value falls outside a predetermined range for systolic pulmonary arterial pressure (sPAP) on TTE, diffusing capacity of the lung for carbon monoxide (DLCO) corrected for haemoglobin, and/or forced vital capacity (FVC) according to the screening algorithm, an email to the treating physician is automatically generated, indicating why the alert has been triggered and recommending a course of action. If the physician chooses not to follow the algorithm he/she must justify this decision.

### Adherence to published recommendations for annual PAH screening

Non-ASIG physicians’ screening practices and adherence to published recommendations for PAH screening [24, 28, 29] were assessed by means of a cross-sectional survey (Additional file 1: Table S1 using survey monkey circulated through the Australian Rheumatology Association following the establishment of ASCS in 2007. The survey addressed existing screening practices based on SSc disease subtype and disease duration (early or late defined as <10 or ≥10 years from the first non-Raynaud’s disease manifestation, respectively), barriers to screening encountered by physicians and suggestions for streamlining screening.

### Statistics

For the purpose of statistical analysis, de-identified aggregated data are exported in.txt and.xml format. Characteristics of patients in the study are presented as mean (standard deviation (SD)) or number (percentage). We compared dichotomous variables using the chi-square test. Continuous variables were compared using the *t* test for normally distributed data and the Wilcoxon rank-sum (Mann-Whitney) test for non-parametric data. All-cause mortality was used for survival analyses. Kaplan-Meier (K-M) curves were used to estimate survival in patients with SSc-PAH diagnosed at first screen versus subsequent screen. The date of PAH diagnosis on RHC was considered the baseline from which survival was calculated. Log-rank and Wilcoxon tests were used to compare survival curves.

## Results

### Recruitment

A total of 1636 patients were recruited into ASCS from July 2007 to July 2016 (census date). Recruitment comprised new referrals from general practitioners for comprehensive specialist management, and from rheumatologists requesting the PAH screening service only. The number of patients recruited, relative to the predicted prevalence of SSc in Australia [31, 32] and population data from the Australian Bureau of Statistics [33] were estimated.

### Patient characteristics and follow up

Of the 1636 patients, 1243 (75.9%) had been seen within the 18 months before the census date and were considered “current” (Table 1). The period of 18 months was selected to allow for delays in data entry. Patient characteristics and follow-up status are summarised in Table 1.

**Table 1:** Characteristics of patients recruited up to July 2016 (n = 1636)

Characteristic	Number (%) or mean ± SD
Patient number	1636
Gender (female:male)	6.4:1 (86.1% vs 13.9%)
Race	
Caucasian	1390 (91.9%)
Asian	75 (4.9%)
Aboriginal-Islander	18 (1.2%)
Hispanic	11 (0.7%)
Other	19 (1.3%)
Age at recruitment, mean ± SD, years	57.2 ± 12.8
Disease duration at recruitment^a^, years	10.9 ± 10.2
Recruited within 4 years of first non-Raynaud’s feature	435
Age at diagnosis of PAH if present, years	62.9 ± 11.1
Number of study visits	
One	364 (22.3%)
Two	325 (19.9%)
Three	216 (13.2%)
Four	199 (12.2%)
Five	165 (10.1%)
Six	119 (7.3%)
Seven	71 (4.3%)
Eight	79 (4.8%)
Nine	84(5.2%)
Ten	14 (0.9%)
Duration of follow up, mean ± SD, years	3.7 ± 2.7
Patient status	
Current	1243 (76.5%)
Withdrawn	101 (6.2%)
Dead	220 (13.5%)
Lost to follow up	61 (3.8%)
Disease subtype	
Limited	1122 (68.6%)
Diffuse	377 (23.0%)
MCTD	83 (5.3%)
Autoantibody profile	
ANA positive (n = 1508)	1418 (94.0%)
Anti-centromere (n = 1497)	673 (44.9%)
Anti-Scl70 (n = 1483)	205 (13.8%)
Anti-RNAP (n = 794)	125 (13.1%)

^a^Disease duration from first non-Raynaud manifestation. Numbers of variables analysed (n =) are included in each section of the table to acknowledge any missing data. Patient status: current patients were defined as being seen in the last 2 years, withdrawn patients have withdrawn their consent from participating in the database and lost to follow up is defined as patients who, despite multiple attempts, we have been unable to contact for >18 months. *PAH* pulmonary arterial hypertension, MCTD mixed connective tissue disease, *ANA* antinuclear antibody, *Anti-RNAP* anti-RNA polymerase

Patients with limited disease (lcSSc) were older at recruitment than patients with diffuse disease (dcSSc) (58.8 ± 12.2 vs 53.1 ± 13.1 years, *p* < 0.001) with longer disease duration from first non-Raynaud’s clinical manifestation (12.2 ± 10.8 vs 8.1 ± 8.7 years, *p* < 0.001). ILD was more prevalent in the patients with dcSSc (40.9% vs 21.1%) but there was no significant difference in the prevalence of PAH (10.1% vs 12.7%, *p* = 0.40) (Table 2).

**Table 2:** Characteristics of patients with SSc by disease subset

	Limited	Diffuse	*P* value
	n = 1122	n = 377	
	mean ± SD or %	mean ± SD or %	
Age at recruitment, years	58.8 ± 12.2	53.1 ± 13.1	<0.001
Female	89.5%	74.9%	<0.001
Disease duration^a^ (non-Raynaud) at recruitment, years	12.2 ± 10.8	8.1 ± 8.7	<0.001
Anti-centromere pattern ANA	59.2%	9.6%	<0.001
Scl 70 positive	8.9%	31.1%	<0.001
RNA polymerase III positive	5.4%	39.6%	<0.001
ILD (HRCT scan)	236 (21.11%)	154 (40.9%)	<0.001
PAH (RHC)	142 (12.7%)	38 (10.1%)	0.40
Rodnan skin score (highest ever)	7.7 ± 5.4	22.7 ± 9.9	<0.001
Digital ulcers ever	39.6%	60.8%	<0.001
Joint contractures ever	28.5%	71.2%	<0.001
Renal crisis ever	0.9%	7.7%	<0.001
Gastro-oesophageal reflux	80.3%	82.7%	0.29
Anal incontinence	27.9%	25.6%	0.39

*ANA* antinuclear antibodies, *ILD* interstitial lung disease, *HRCT* high-resolution chest computed tomography, *PAH* pulmonary arterial hypertension, *RHC* right-heart catheterisation

^a^Disease duration defined as from first non-Raynaud’s disease manifestation. Disease manifestations are defined as present if ever present from time of diagnosis of systemic sclerosis (SSc)

In total, 232 patients (14.2%) were diagnosed with pulmonary hypertension (PH). Of these, 194 patients (83.6%) had World Health Organisation (WHO) Group I PAH according to the criteria developed in Nice [34], 15 patients (6.5%) had exercise-induced PH, 18 patients (7.8%) had PH secondary to left ventricular dysfunction (WHO Group 2 PH) and 5 patients (2.2%) had PH secondary to ILD (WHO Group 3 PH). Only patients with WHO Group I PAH were analysed in this study.

At enrollment, 34 (2.1%) patients had previously been diagnosed with PAH and 122 (7.5%) were diagnosed at their first contact with ASCS. Among the latter, 89 (72.9%) had SSc disease duration of ≥4 years and the date of their last TTE was 1.6 ± 4.6 years before enrollment to ASCS, highlighting a lack of adherence to annual PAH screening in the community.

### Outcomes of screening in the ASCS

A total of 4326 screening visits were analysed, to determine the success of, and adherence to the PAH screening algorithm (Table 3). Only patients not previously diagnosed with PAH were included. At the first screening visit, sufficient data to apply the screening algorithm were available for 1363 patients. Of these, 101 patients (7.4%) were at high risk of PAH, 121 patients (8.9%) at moderate risk, 358 patients (26.3%) at low risk and 470 patients (34.5%) at no increased risk based on TTE and PFT results. The number of patients with no tricuspid regurgitation (TR) on TTE, and hence inestimable sPAP, was high at 313 (22.9%).

**Table 3:** Diagnosis of WHO Group I PAH by risk stratification using the screening algorithm

		No increased probability^a^			Low probability of PAH^a^			Moderate probability of PAH^a^			High probability of PAH^a^			Missing sPAP			Total with PAH, *n*
Review number	Screened, *n*	Normal TTE, *n*	RHC, *n*	PAH, *n*	Moderate TTE, *n*	RHC, *n*	PAH, *n*	High suspicion TTE, *n*	RHC, *n*	No. with PAH	High probability TTE, *n*	RHC, *n*	PAH, *n*	Positive screen^c^, *n*	RHC, *n*	PAH, *n*	
1st screen	1363	470	19	4	358	38	11	121	36	25	101	83	72	313	24	10	122 (8.9%)
2nd screen	896	355	6	2	229	5	3	78	10	4	32	11	5	202	8	3	17 (1.9%)
3rd screen	679	272	0	0	167	4	1	65	5	3	20	7	6	155	3	1	11 (1.6%)
4th screen	482	199	0	0	125	3	0	42	7	2	13	5	3	103	1	0	5 (1.0%)
5th screen	361	149	0	0	104	0	0	25	1	1	9	4	1	74	1	0	2 (0.6%)
6th screen	235	91	0	0	72	1	1	25	1	1	6	0	0	41	0	0	2 (0.9%)
7th screen	162	66	0	0	48	0	0	14	0	0	3	0	0	31	1	0	0 (0.0%)
8th screen	104	48	0	0	27	1	1	10	0	0	1	0	0	18	0	0	0 (0.0%)
9th screen	43	20	0	0	11	1	1	6	0	0	1	0	0	5	0	0	1 (2.3%)
10th screen	1	0	0	0	1	0	0	0	0	0	0	0	0	0	0	0	0 (0.0%)
Total	4326	1670	25^b^ (1.5%)	6^a^ (24%)	1142	53^b^ (4.6%)	17^a^ (32.7%)	386	60^b^ (15.5%)	36^a^ (60%)	186	110^b^ (59%)	87^a^ (79.1%)	942	38^b^ (4%)	14^a^ (36.8%)	160 (11.7%)

^a^Probability of pulmonary arterial hypertension (PAH): no increased probability of PAH was indicated by systolic pulmonary arterial pressure (sPAP) <30 and diffusing capacity of the lung for carbon monoxide corrected for haemoglobin (DLCO) >50%, low probability of PAH was indicated by 30 ≤ sPAP <40 mmHg with normal forced vital capacity (FVC) and high-resolution computed tomography (HRCT), moderate probability of PAH was 40 ≤ sPAP <50 mmHg OR DLCO ≤50% and FVC <85% or annual fall in DLCO ≥20% or unexplained dyspnea and high probability of PAH was indicated by sPAP ≥50 mmHg.

^b^Percentage of patients who were detected by transthoracic echocardiogram (TTE) to be in a risk category and underwent right-heart catheterisation (RHC)

^c^Percentage of patients who underwent RHC and were diagnosed with World Health Organisation (WHO) Group I PAH

At the first screening visit, 157 of the 580 (27.1%) patients at low (38/358), moderate (36/121), or high (83/101) risk of PAH underwent RHC. WHO Group 1 PAH was diagnosed in 122 patients (8.9% of the cohort), including 4 of 19 (21.0%) patients with a normal TTE and 10 of 24 patients (41.7%) with a missing sPAP on TTE due to lack of a TR jet. The patients in the latter two groups were referred for RHC due to reduced exercise capacity and/reduced DLCO. Only 4% of the patients with no TR were referred for RHC but 36.8% of these were diagnosed with WHO Group 1 PAH. At the physician’s discretion and based on other indicators such as patient symptoms and TTE parameters, patients whose initial RHC was negative for PAH were considered for future RHC.

The screening algorithm correctly identified patients at increased risk of PAH and stratified them by level of risk. The higher the probability of PAH, the more likely PAH was to be identified on RHC (Table 3). A new diagnosis of WHO Group I PAH was made in 160 patients from 4326 (3.7%) screening visits (115/1122 with lcSSc, 32/377 with dcSSc and 10/83 with MCTD), giving a prevalence of PAH of 11.8% (10.3% in lcSSc, 8.5% in dcSSc and 12.0% in MCTD). The annual prevalence of PAH was 1.4% (1.2% in lcSSc, 0.9% in dcSSc, 0.4% in MCTD). All patients were treated with specific PAH therapy.

### Characteristics of patients with PAH

Patients with PAH were significantly older than patients without PAH (63.1 vs 56.3 years, *p* < 0.001), had longer disease duration (13.6 vs 10.4 years, *p* < 0.001) and were in WHO Functional Class III/IV (85.0% vs 21.7%, *p* < 0.001) (Table 4). Clinical manifestations and autoantibody status are summarised in Table 4.

**Table 4:** Characteristics of patients with SSc by PAH status

	PAH	No PAH	*P* value
	mean ± SD or %	mean ± SD or %	
Number of patients	209	1283	
Age at recruitment, years	63.1 ± 10.4	56.3 ± 12.7	<0.001
Disease duration at recruitment, years^a^	13.6 ± 11.5	10.4 ± 9.9	<0.001
Female	87.1%	86.2%	0.88
Limited disease subtype	73.9%	70.3%	0.43
Anti-centromere pattern ANA	52.6%	44.1%	0.02
Scl 70 positive	6.9%	15.2%	0.003
RNA polymerase III positive	14.4%	12.9%	0.65
Digital ulcers ever	53.0%	41.9%	0.003
Telangiectasia ever	91.4%	82.4%	<0.001
Calcinosis ever	53.3%	34.7%	<0.001
Joint contractures ever	45.8%	35.7%	0.007
GORD	45.9%	45.3%	0.48
Bowel dysmotility	27.3%	22.7%	0.15
Anal incontinence	32.1%	25.7%	0.05
WHO Functional Class			
Class I	4 (2.1%)	467(39.1%)	<0.001
Class II	25 (12.9%)	469 (39.2%)	
Class III	106 (54.9%)	238 (19.9%)	
Class IV	58(30.1%)	22(1.8%)	
NT-pro-BNP	218.9 ± 285.7	75.1 ± 159.8	0.005

*PAH* pulmonary arterial hypertension, ANA antinuclear antibodies, *6MWD* six minute walk distance, *mPAP* mean pulmonary arterial pressure, *mRAP* mean right atrial pressure, *PVR* pulmonary vascular resistance, *GORD* gastro-oesphageal reflux disease, *WHO* World Health Organisation, *NT-pro-BNP* N-terminal pro b-type natriuretic peptide

^a^Disease duration defined as from first non-Raynaud’s disease manifestation

Disease manifestations defined as present if ever present from systemic sclerosis (SSc) diagnosis

### Characteristics of patients with PAH diagnosed on first screen compared with patients whose PAH was diagnosed on subsequent screens

To assess whether the ASCS screening programme succeeded in detecting PAH at an earlier stage, we divided our PAH cohort into those patients whose PAH was detected on first screening and those whose PAH was detected on the second or subsequent screen (Table 5).

**Table 5:** Comparison of demographic, clinical and haemodynamic characteristics of patients with PAH diagnosed at first screen and PAH diagnosed at subsequent screens

Characteristic	PAH detected on first screening	PAH detected on subsequent screening	*P* value
	mean ± SD or %	mean ± SD or %	
Number of patients	122	38	
Age at PAH diagnosis	63.9 ± 11.0	62.7 ± 9.5	0.56
Disease duration at PAH diagnosis	13.4 ± 12.8	14.6 ± 8.7	0.62
Female	96 (85.7%)	32 (84.2%)	0.82
Disease subtype			
Limited	81 (75.7%)	23 (62.2%)	0.03
Diffuse	17 (15.9%)	13 (35.1%)	
MCTD	9 (8.4%)	1 (2.7%)	
Status			
Alive	54 (48.2%)	26 (68.4%)	0.03
Dead	55 (49.1%)	10 (26.3%)	
Withdrawn	3 (2.7%)	1 (2.6%)	
Unable to contact	0 (0%)	1 (2.6%)	
Follow up, years	3.3 ± 2.4	5.9 ± 1.9	<0.001
WHO class at PAH diagnosis			
Class I	4 (4.0%)	0 (0%)	0.01
Class II	16 (16.2%)	15 (40.5%)	
Class III	63 (75.9%)	20 (54.1%)	
Class IV	16 (16.2%)	2 (5.4%)	
6MWD at PAH diagnosis	295.9 ± 118.3	340.6 ± 115.6	0.05
mPAP on RHC	38.2 ± 11.4	32.5 ± 8.3	0.005
mRAP on RHC	9.7 ± 8.5	8.4 ± 3.9	0.40
PVR on RHC	5.5 ± 3.1	3.8 ± 1.7	0.005
CI on RHC	2.9 ± 1.6	2.9 ± 0.7	0.87
Pericardial effusion	16 (16.0%)	1 (2.7%)	0.03

Probability of PAH

*PAH* pulmonary arterial hypertension, *MCTD* mixed connective tissue disease, *WHO* World Health Organisation, *6MWD* six-minute walk distance, *mPAP* mean pulmonary arterial pressure, *RHC* right-heart catheterization, *mRAP* mean right atrial pressure, *PVR* pulmonary vascular resistance, *CI* cardiac index, *NT-pro-BNP* N-terminal pro b-type natriuretic peptide

We believe that PAH detected on first screen were likely to be a “delayed diagnosis” in patients who were referred to ASCS due to symptoms or clinical suspicion of PAH, whereas PAH detected on subsequent screens was more likely to be an “incident” PAH identified on earlier screening.

Patients diagnosed with PAH on subsequent screens compared with first screen were more likely to have dcSSc (35.1% vs 15.9%, *p* = 0.03), be in a better WHO functional class (54.1% vs 75.9% in Class III, 5.4% vs 16.2% in Class IV, *p* = 0.01) and have less advanced PAH at diagnosis evidenced by lower mPVR (3.8 vs 5.5, *p* = 0.005), higher mean 6MWD (340.6 vs 295.9, *p* = 0.05), lower mPAP (32.5 vs 38.2, *p* = 0.005), and fewer non-trivial pericardial effusions (2.7% vs 16.0%, *p* = 0.03).

**Fig. 2:**
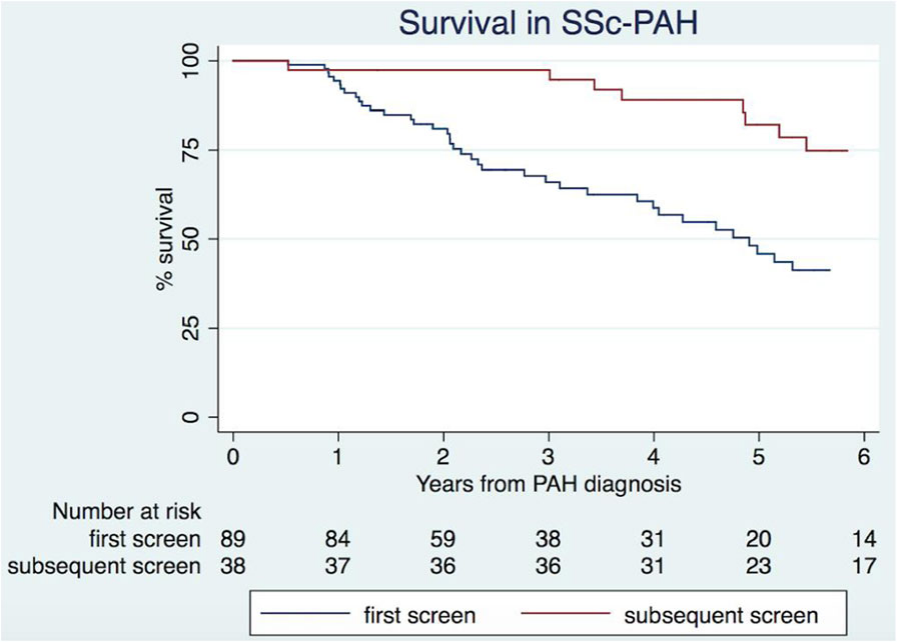
Survival in systemic sclerosis (*SSc*) with pulmonary arterial hypertension (*PAH*) according to the screening visit when the diagnosis was made (first vs subsequent)

PAH treatment with combination therapy and anticoagulation therapy was prescribed at a similar rate in those diagnosed at subsequent screens (36.8% and 28.9%, respectively) and those diagnosed at first screen (32.1% and 26.8%, respectively). Three-year survival from the time of PAH diagnosis was better in those diagnosed on subsequent screen compared with those diagnosed at first screen as outlined in Fig. 2 (94.7% vs 42.7%, *p* < 0.001) and mean time to death was longer in those diagnosed with PAH on subsequent screens compared with those diagnosed at first screen (4.7 ± 2.3 vs 2.3 ± 2.3, *p* < 0.001). PAH was the direct cause of death in 100% of patients diagnosed on subsequent screen and in 92.7% of those diagnosed on first screen (*p* = 0.40).

### Adherence to the ASCS screening algorithm

ASIG physicians’ adherence to PAH screening guidelines was high with 84.4% of patients undergoing annual screening over a ten-year period. Despite the screening algorithm (Fig. 1) mandating that patients at moderate or high risk of PAH should undergo RHC, only 29.7% (170/572) had RHC. Even among those in the high probability group, only 59.1% of patients underwent RHC. Physician justification for not performing RHC predominantly related to their WHO functional class. In 50.5% of cases, RHC was not performed as the patient was in WHO Functional Class I (31.6%), or Class II (14.7%) or was not dyspnoeic (4.2%). In 18.9% of cases, the physician referred the patient to another specialist for their opinion, most commonly a cardiologist or respiratory physician. In 16.8% of cases, the physician was deterred from arranging RHC due to the presence of mild ILD that they felt accounted for the TTE or PFT result. The remaining justifications included a plan to repeat the TTE and/or PFT at a later date (4.2%), patient refusal to have RHC (2.1%), terminal malignancy (2.1%) and no access to RHC (1.1%).

### Adherence to PAH screening guidelines by Australian rheumatologists after the establishment of the ASCS screening programme in 2007

Of 334 non-ASIG Australian rheumatologists, 52 responded to the survey on screening practices and barriers to screening (Additional file 1: Table S1). The majority (88.9%) reported screening asymptomatic patients for PAH regularly. Just over half of the respondents ordered annual screening for their asymptomatic patients with early SSc (lcSSc 58.9%, dcSSc 52.6%), with even fewer screening annually in late disease (lcSSc 38.5%, dcSSc 43.6%). All respondents (100%) would investigate their patients with symptoms consistent with PAH. In early symptomatic SSc (with breathlessness or reduced exercise tolerance), the majority of respondents would screen on a six-monthly (51.2%) or annual basis (33.6%). In late symptomatic SSc, 38.9% of respondents would screen their patients six-monthly and 41.2% of respondents would screen annually. Alarmingly, 17.5% of respondents would only screen symptomatic patients every two years.

Explanations for not following guidelines for PAH screening included cost of screening (60%) and concerns about how to interpret the results (80%). In order to improve screening, 50% of respondents felt that they required better guidelines for the selection and frequency of screening tests, 44.7% wanted a reminder system, 42.1% wanted guideline simplification but only 31.6% felt that access to an experienced screening centre would be helpful. If reimbursed by Medicare Australia, 76.3% of respondents would consider the use of a blood test such as N-terminal pro B-type natriuretic peptide (NT-pro-BNP) a major advance.

## Discussion

We present the ASCS as a model of multi-centre web-based data collection and decision support for applying a PAH screening algorithm in patients with SSc. Between 2007 and 2016, 1636 patients with SSc were recruited, an estimated 30.9% of all Australian patients with SSc based on a prevalence of 20 per 100,000 people. As Australia has such sparse population density and SSc is a low-frequency disease, such multicentre collaborations are required in order to recruit and retain sufficient patients for a longitudinal observational study.

The patient characteristics of our cohort are comparable to those of other large multi-centre cohorts indicating that a representative sample is being recruited [35]. As a result of screening, 160 patients were diagnosed with PAH over a ten-year period, providing an annual PAH of 1.4%, consistent with that of the French *ItinerAIR-Sclerodermie* Study of 0.6% [36] and a prevalence of 11.8% consistent with worldwide data [13, 36–39].

As in other cohorts [40, 41], patients with PAH were older, had longer disease duration from the first non-Raynaud’s clinical manifestation, were more likely to be anti-centromere (ACA)-positive and to have digital ulcers, telangiectasia, calcinosis and joint contractures compared to those without PAH. Patients with PAH detected on the second or subsequent screen were more likely to have dcSSc, be in a better WHO Functional Class at PAH diagnosis and have less advanced PAH, which is consistent with the French data [36] but different from the classical teaching that PAH more commonly develops in patients with lcSSc [42].

Additionally, patients with PAH diagnosed on subsequent screen compared with first screen had significantly improved survival (*p* < 0.001) and a longer mean time to death (4.7 ± 2.3 vs 2.3 ± 2.3, *p* < 0.001), which may in part be due to lead-time bias. Our one-year and three- year survival in patients diagnosed with PAH on subsequent visits are similar to the survival rates in the PHAROS registry [14], one of the few other studies evaluating survival in incident SSc-PAH diagnosed on RHC. These survival rates are higher than those reported in other registries containing a mixture of patients with both incident and prevalent SSc-PAH or PAH diagnosed on TTE rather than RHC [38, 43, 44]. Diagnosis of early PAH is particularly important as patients with early PAH can progress rapidly, supporting the need for early treatment in this patient population [12].

Despite the majority of patients in the ASCS being screened annually, only 29.7% (170/572) of patients deemed at moderate or high risk of PAH were referred for RHC, predominantly due to preservation of functional class limiting patient eligibility for PBS-funded PAH therapy. Concern about the small but documented risk involved in RHC may also have deterred physicians from referring for RHC but this information was not specifically sought. These results highlight external factors that limit adherence to international screening recommendations.

The number of patients with no TR on TTE, and hence inestimable sPAP, was high at 21.8%, but consistent with the 20–30% reported in the literature [45]. Only 4% of these patients were referred for RHC, with 36.8% diagnosed with WHO Group 1 PAH. This highlights the need for a non-TTE dependent method of PAH screening and supports the emerging role of NT-pro-BNP, which is increased in PAH and in those at increased risk [46]. This test is not currently reimbursed for this indication in Australia and thus is not often performed.

Since the establishment of ASCS in 2007, Australian rheumatologists’ adherence to screening guidelines has improved, with 88.9% of physicians regularly screening asymptomatic patients with SSc for PAH compared with 55.8% previously [29]. Of those who did not perform TTE for PAH screening, 50% reported this was due to difficulty assessing the right heart pressures with TTE. Referral of these patients to a tertiary screening centre may overcome this obstacle. To our knowledge, there have been no other studies addressing physicians’ perceived barriers to SSc-PAH screening.

Limitations to our study include the potential for lead-time and length-time bias to impact on the survival benefit seen in PAH detected with screening. However, patients detected by screening were functionally impaired at the time of diagnosis and were all commenced on PAH therapy, indicating that their physician felt that their PAH required treatment. Our data analyses were conducted in patients who underwent all procedures listed in our screening algorithm; therefore, the incidence of PAH in our study may be an underestimate, although consistent with the literature. Additionally, only 15.6% of non-ASIG Australian rheumatologists responded to our survey on PAH screening adherence. Therefore these particular results may not be a true reflection of rheumatologists nationwide.

## Conclusion

SSc-PAH is a tragic consequence of SSc, which despite advances in therapy, is the leading cause of SSc-related death. Screening with a web-based algorithm can identify patients with earlier PAH and improve outcomes. Identifying modifiable barriers to screening may improve adherence and ultimately patient outcomes.

## Additional file

Additional file 1: Table S1. Cross-sectional survey of physician adherence to PAH screening recommendations. (DOC 97 kb)

## Abbreviations

ASCS: Australian Scleroderma Cohort Study
ASIG: Australian Scleroderma Interest Group
DLCO: Diffusing capacity of the lung for carbon monoxide corrected for haemoglobin
dcSSc: Diffuse cutaneous systemic sclerosis
HRCT: High-resolution computed tomography
ILD: Interstitial lung disease
lcSSc: Limited cutaneous systemic sclerosis
MCTD: Mixed connective tissue disease
NT-pro-BNP: N-terminal pro B-type natriuretic peptide
PAH: Pulmonary arterial hypertension
PH: Pulmonary hypertension
RFTs: Respiratory function tests
RHC: Right-heart catheterisation
SSc: Systemic sclerosis (also known as systemic scleroderma)
sPAP: Systolic pulmonary arterial pressure
TR: Tricuspid regurgitation
TTE: Transthoracic echocardiogram
WHO: World Health Organisation
