# Supplementary material for: Epidemiology and disease characteristics of systemic sclerosis-related pulmonary arterial hypertension: results from a real-life screening programme
Source: Arthritis Res Ther. 2017 Mar 7;19:42. doi: 10.1186/s13075-017-1250-z (PMC5341425; doi:10.1186/s13075-017-1250-z)
Supplement: Additional file 1: Table S1. — Cross-sectional survey of physician adherence to PAH screening recommendations. (DOC 97 kb) [file 13075_2017_1250_MOESM1_ESM.doc]

Supplementary Table 1: Cross-sectional survey of physician adherence to PAH screening recommendations

Q1. In which state or territory do you mainly practice?

| Australian Capital Territory | 0% |
| --- | --- |
| New South Wales | 42.3% |
| Northern Territory | 0% |
| Queensland | 9.6% |
| South Australia | 9.6% |
| Tasmania | 5.8% |
| Victoria | 26.9% |
| Western Australia | 5.8% |

Q2. What proportion of your patients do you see in the following locations?

| Inner City | 50% |
| --- | --- |
| Suburban | 67% |
| <100km from the CBD of a large city | 34% |
| >100km from the CBD of a large city | 25% |

# Q3. Please indicate the proportions (%) of your time you spend in the following:

| Private practice | 57% |
| --- | --- |
| Hospital practice with a designated PAH centre | 36% |
| Hospital practice without a designated PAH centre | 30% |
| In research | 16% |
| In teaching | 13% |
| In other activities | 12% |

# Q4. Do you work in a centre where PBS - funded therapies for pulmonary arterial hypertension (PAH) eg bosentan, ambrisentan can be prescribed?

| Yes | 59.6% |
| --- | --- |
| No | 40.4% |

# Q5. Please estimate the number of patients you have in your practice with systemic sclerosis:

| 0 | 2.1% |
| --- | --- |
| 0-5 | 19.2% |
| 5-10 | 31.9% |
| 10-50 | 28.3% |
| >50 | 8.5% |

# Q6. Do you screen your patients with systemic sclerosis who have NO breathlessness or reduced exercise capacity, for pulmonary arterial hypertension (PAH) on a REGULAR basis, assuming they remain stable?

| Yes | 88.9% |
| --- | --- |
| No | 11.1% |

# Q7. How often do you screen patients with systemic sclerosis with NO breathlessness or reduced exercise capacity, for PAH? “Late” implies >10 years of disease since the first non-Raynaud’s symptom.

| SSc category | Every 3 months | Every 6 months | Annually | Every 2 years | Only if symptomatic |
| --- | --- | --- | --- | --- | --- |
| Early limited SSc | 5.1% | 7.7% | 58.9% | 25.6% | 2.6% |
| Late limited SSc | 5.1% | 7.7% | 38.5% | 38.5% | 10.3% |
| Early diffuse SSc | 7.9% | 15.8% | 52.6% | 18.4% | 5.3% |
| Late diffuse SSc | 5.1% | 2.6% | 43.6% | 38.5% | 10.3% |

# Q8. Please rate the importance of each of the following reasons for not regularly screening SSc patients with NO breathlessness or reduced exercise capacity, for PAH

|  | Not at all important | Not really important | Neither important nor unimportant | Somewhat important | Very important |
| --- | --- | --- | --- | --- | --- |
| No access to good quality services for investigations | 80% | 0% | 20% | 0% | 0% |
| Cost for the patient | 40% | 0% | 0% | 40% | 20% |
| Cost for the health service | 20% | 0% | 20% | 40% | 20% |
| Unsure how to interpret the results | 20% | 0% | 0 | 80% | 0% |
| I would refer to a screening centre, but lack information on how to access these centres | 40% | 20% | 40% | 0% | 0% |
| I would prefer to refer to a screening centre, but too far away | 60% | 0% | 40% | 0% | 0% |
| Screening is not useful in these patients | 20% | 0% | 20% | 20% | 40% |
| Screening is not proven to be cost-effective in these patients | 0% | 20% | 40% | 20% | 20% |
| No advantage in identifying PAH until these patients have symptoms | 20% | 20% | 20% | 20% | 20% |

# Q9. Do you screen your patients with systemic sclerosis who HAVE breathlessness or reduced exercise capacity, for PAH on a REGULAR basis, assuming they remain stable?

| Yes | 100% |
| --- | --- |
| No | 0% |

# Q11. How often do you screen patients with systemic sclerosis who HAVE breathlessness or reduced exercise capacity, for PAH?

|  | Every 3 months | Every 6 months | Annually | Every 2 years |
| --- | --- | --- | --- | --- |
| Early limited SSc | 6.9% | 51.2% | 32.6% | 9.3% |
| Late limited SSc | 11.6% | 51.2% | 34.9% | 2.3% |
| Early diffuse SSc | 9.3% | 34.9% | 41.8% | 13.9% |
| Late diffuse SSc | 7.1% | 42.8% | 40.5% | 9.5% |

# Q12. Which of the following investigations do you usually request in systemic sclerosis patients with or without symptoms as SCREENING tests for PAH

| Chest XRay | 51.2% |
| --- | --- |
| ECG | 48.8% |
| Pulmonary function test | 90.2% |
| HRCT scan chest | 31.7% |
| Doppler ECHO | 87.8% |
| Stress ECHO | 9.8% |
| Right heart catheter | 2.4% |
| Brain natriuretic peptide or NT-pro-BNP | 4.9% |
| Six minute walk test | 24.4% |

# Q13. If you do not use Doppler echocardiogram (ECHO) as a PAH screening tool, please rate the importance of each of the following reasons why

|  | Not at all important | Not really important | Neither important nor unimportant | Somewhat important | Very important |
| --- | --- | --- | --- | --- | --- |
| Difficulty accessing ECHO service that assesses right heart adequately | 50% | 0% | 25% | 25% | 0% |
| Not obtaining an estimate of the systolic pulmonary arterial pressures in every patient | 50% | 0% | 25% | 25% | 0% |
| The nearest centre offering ECHO is too far away | 50% | 0% | 50% | 0% | 0% |
| Cost of ECHO study for the patient | 50% | 0% | 50% | 0% | 0% |
| Cost of ECHO study for the health service | 66.7% | 0% | 33.3% | 0% | 0% |
| Unsure how to interpret an ECHO study | 33.3% | 33.3% | 33.3% | 0% | 0% |

# Q 14 How useful do you find published guidelines for screening for PAH?

| Very helpful | 28.9% |
| --- | --- |
| A bit helpful | 42.1% |
| Neither helpful or unhelpful | 23.7% |
| A bit unhelpful | 0% |
| Very unhelpful | 5.3% |

# Q15. Which of the following, would you find helpful in streamlining the screening process for cardio-pulmonary complications in SSc patients?

| Improved guidelines for selection and frequency of screening tests | 50% |
| --- | --- |
| A reminder system for screening tests based on guidelines | 44.7% |
| Access to a centre with experience in screening patients | 31.6% |
| Simplification of screening guidelines | 42.1% |

# Q16. If a validated blood test costing around $50 were available for screening for PAH, please rate how acceptable this would be to you as an alternative to ECHO as a first line screening test (this does not exclude ECHO if the screening tests are positive or it is clinically indicated)?

|  | Totally unacceptable | Not ideal but might consider it under certain circumstances | Neither acceptable nor unacceptable | Acceptable in most circumstances | A major advance |
| --- | --- | --- | --- | --- | --- |
| If reimbursed by medicare | 2.6% | 2.6% | 2.6% | 15.8% | 76.3% |
| If the patient incurred the cost | 7.9% | 18.4% | 7.9% | 44.7% | 21.1% |
| If the health service incurred the cost | 5.3% | 2.6% | 0% | 39.5% | 52.6% |
